# An unbiased metagenomic search for infectious agents using monozygotic twins discordant for chronic fatigue

**DOI:** 10.1186/1471-2180-11-2

**Published:** 2011-01-02

**Authors:** Patrick F Sullivan, Tobias Allander, Fredrik Lysholm, Shan Goh, Bengt Persson, Andreas Jacks, Birgitta Evengård, Nancy L Pedersen, Björn Andersson

**Affiliations:** 1 Department of Genetics, University of North Carolina at Chapel Hill, NC, USA; 2 Department of Medical Epidemiology and Biostatistics, Karolinska Institutet, Stockholm, Sweden; 3 Laboratory for Clinical Microbiology, Department of Microbiology, Tumor and Cell Biology, Karolinska University Hospital, Karolinska Institutet, Stockholm, Sweden; 4 Department of Cell and Molecular Biology, Science for Life Laboratory, Karolinska Institutet, Stockholm, Sweden; 5 IFM Bioinformatics, Linköping University Linköping, Sweden; 6 Department of Clinical Microbiology, University of Umeå, Umeå, Sweden

## Abstract

**Background:**

Chronic fatigue syndrome is an idiopathic syndrome widely suspected of having an infectious or immune etiology. We applied an unbiased metagenomic approach to try to identify known or novel infectious agents in the serum of 45 cases with chronic fatigue syndrome or idiopathic chronic fatigue. Controls were the unaffected monozygotic co-twins of cases, and serum samples were obtained at the same place and time.

**Results:**

No novel DNA or RNA viral signatures were confidently identified. Four affected twins and no unaffected twins evidenced viremia with GB virus C (8.9% vs. 0%, p = 0.019), and one affected twin had previously undetected hepatitis C viremia. An excess of GB virus C viremia in cases with chronic fatigue requires confirmation.

**Conclusions:**

Current, impairing chronic fatigue was not robustly associated with viremia detectable in serum.

## Author Summary

The cause of chronic fatigue syndrome is unknown but infections with viruses have been suspected. We used a new approach to screen blood samples for the presence of known or novel viral infections. Samples were 45 cases with chronic fatigue syndrome or idiopathic chronic fatigue, and controls were their unaffected monozygotic co-twins. No novel DNA or RNA viral signatures were confidently identified. Four affected twins and no unaffected twins evidenced viremia with GB virus C (8.9% vs. 0%, p = 0.019), and one affected twin had previously undetected hepatitis C viremia. An excess of GB virus C viremia in cases with chronic fatigue requires confirmation. However, current, impairing chronic fatigue was not robustly associated with viral infections in serum detectable by our methods.

## Background

Chronic fatigue syndrome (CFS) is characterized by prolonged and impairing fatigue of unknown etiology [1,2]. The standard definition of CFS requires severe fatigue of over six months duration that remains unexplained despite appropriate clinical medical evaluation along with four of eight signs and symptoms (e.g., post-exertional malaise and impaired memory or concentration). Immune dysfunction is a major etiological hypothesis, and could result from a chronic infection or an inappropriate response to an initial infection [3-7]. Multiple studies have investigated the possible role of a range of specific viruses in CFS by searching for case-control differences in past or current viral infection (e.g., cytomegalovirus, Epstein-Barr virus, hepatitis C, human herpes virus-6, and parvovirus B19) [5]. Inconsistent findings across studies are normative. The most recent example is xenotropic murine leukemia virus-related virus (XMRV) which was claimed to be present in 67% of cases with CFS and 3.7% of controls [8] but did not replicate in multiple independent samples [9]. A recent report found an association between a different retrovirus (murine leukemia virus) and CFS (87% of cases, 7% of controls) [10]. The status of any connection between XMRV and CFS is remains highly controversial [11].

It is possible that the etiology of CFS is not unitary. Non-replication across samples would be expected if different combinations of etiological processes were operative in different case sets. Alternatively, inconsistent findings across case-control studies could be due to bias if controls are inappropriate to cases. For example, in the initial XMRV study, cases were highly selected (chronically ill patients treated in medical practices specializing in CFS) and controls were described only as "healthy" [8]. Although such individuals are relatively uncommon, the study of discordant monozygotic twins offers substantially improved experimental control (i.e., an individual affected with CFS and their well monozygotic twin) [12]. We are aware of one previous study that assessed 22 pairs of monozygotic twins discordant for CFS for indices of past and current viral infection (BK virus; cytomegalovirus; Epstein-Barr virus; hepatitis C virus; herpes simplex virus 1 and 2; human herpes virus 6, 7, and 8; JC virus; parvovirus B19; and varicella zoster virus): no significant or clinically important differences were found between affected and unaffected twins [13].

An additional limitation has been the reliance on assays for specific infectious agents. Viruses have traditionally been identified by culture techniques and more recently via a variety of molecular approaches. However, these methods have severe limitations and leave many viruses undetected. We have developed a complete "metagenomic" system for systematic identification of unknown viruses. The discovery pipeline has four components: virus enrichment, amplification of genomic viral DNA or RNA, large scale sequencing, and identification of known and novel viral sequences using bioinformatics. This powerful strategy has identified two new viruses, human bocavirus [14] and KI polyomavirus [15] which cause acute respiratory illness in children.

In this study, 45 pairs of monozygotic twins discordant for chronic fatigue were used in an exhaustive study to identify risk factors [12]. We report here the results of screening for viruses in these samples using metagenomic sequencing. Deep sequencing revealed the presence of several viruses in cases with chronic fatigue, particularly GB virus C.

## Results

The patient set consisted of 45 pairs of monozygotic twins discordant for clinically-evaluated chronic fatiguing illness (Table 1). Most pairs were female (89%), and the median age at evaluation was 51 years. Of the affected twins, 32 met criteria for CFS and 13 for ICF with a median duration of chronic fatigue of 8 years with no significant difference between affected twins with CFS and ICF (p = 0.75). Body mass index was similar between the affected and unaffected twins. Affected twins had significantly worse physical and mental functioning on the SF-36 [16] and reported significantly greater current fatigue. The mean functioning of affected twins was over a standard deviation worse than Swedish norms whereas the unaffected twins were similar to Swedish norms (http://www.sf-36.org/nbscalc/index.shtml, accessed 12 December 2008).

**Table 1:** Description of 45 monozygotic twin pairs discordant for chronic impairing fatigue.

Variable	Affected twins	Unaffected co-twins	Statistical comparison
Met criteria for chronic fatigue syndrome	32/45, 71%	0/45, 0%	*By design*
Met criteria for idiopathic chronic fatigue	13/45, 29%	0/45, 0%	*By design*
Female sex	40/45, 89%	40/45, 89%	*Identical by design*
Median age at evaluation, IQR	51, 39-59 years	51, 39-59 years	*Identical by design*
Median body mass index, IQR	25, 22-30 kg/m^2^	24, 22-31 kg/m^2^	Paired t_44 _= 0.1, p = 0.91
Median SF-36 physical function, IQR	41, 27-48	48, 39-52	Paired t_44 _= 3.1, p = 0.003
Median SF-36 mental function, IQR	39, 29-48	51, 39-56	Paired t_44 _= 4.7, p = 3 × 10^-5^
Median current fatigue by VAS, IQR	69, 49-77	19, 10-51	Paired t_43 _= -7.2, p = 6 × 10^-9^

Abbreviations: IQR = inter-quartile range, VAS = visual analogue scale (0-100).

### Using metagenomic sequencing to identify viral signatures

**Figure 1:**
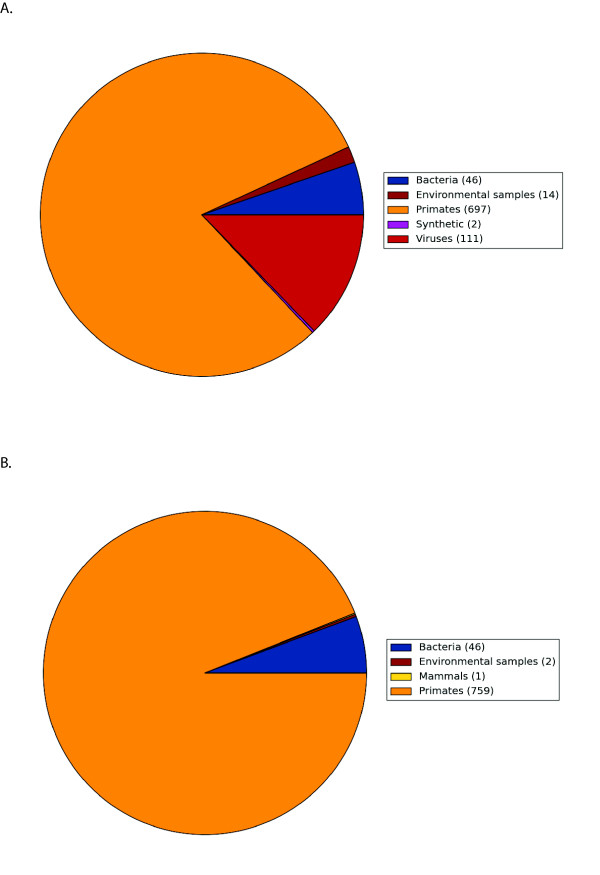
**Comparison of BLAST results from Sanger reads (post-assembly) that were classified with high confidence from twins affected with chronic fatiguing illness (panel A) and their unaffected co-twins (panel B)**. The results show a large viral fraction in affected samples and no viral sequences in unaffected samples.

Serum samples from the affected and unaffected twins were pooled separately and enriched for viral particles. This resulted in four samples to be sequenced in order to detect RNA and DNA viruses: a DNA sample and a cDNA sample for pooled samples from affected and unaffected twins. Sanger sequencing was performed from all four samples, resulting in a total of 1,549 sequences from affected twins and 1,513 from unaffected twins. Automated BLAST searches followed by manual inspection showed that all reads from the unaffected twins were from background contamination (mostly human or bacterial) or from reagents used for the library preparation (Figure 1). A small number of sequences showed no or only insignificant BLAST hits but manual inspection did not reveal any artifacts and these could represent low abundance viral sequences. In contrast, the sequences from the pool of affected twins showed multiple hits to two known human viruses. In total, 168/1,549 sequences showed a significant BLAST identity to GB virus C (GBV-C) and 15/1,549 to hepatitis C virus. The numbers of sequences were relatively high indicating that one or more affected twins had high copy numbers for these viruses. No other significant hits to human viruses were observed.

**Figure 2:**
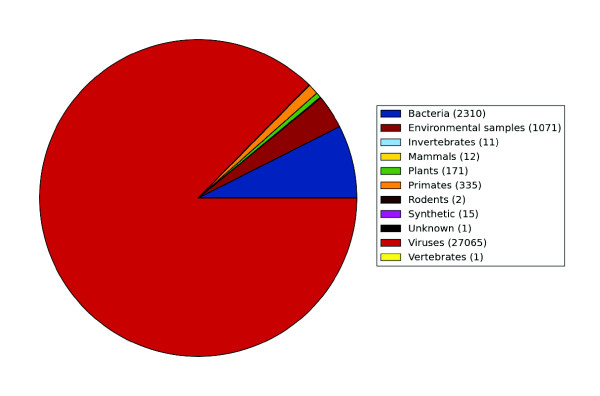
**BLAST results from Roche 454 reads that were classified with high confidence from affected samples after pre-assembly screening removing high confidence human and repetitive sequences**. A large viral fraction can be seen.

A next-generation sequencing technology, Roche 454 FLX, was used to search for rare viruses in samples from affected twins. A total of 53,985 sequence reads (9.1 Mb) were produced from the DNA sample and 305,191 reads (59.5 Mb) from the RNA (+RT) sample. The six-fold difference in the numbers of reads was most likely caused by different efficiencies of the 454 library preparation and the amounts of DNA obtained. The reads were analyzed using our BLAST search pipeline, both unassembled and assembled (together with the Sanger reads after removal of most human sequences) using the miraEST assembler. The assembly results are shown in Tables 2, 3, and 4. The BLAST results are summarized in Figure 2 and Additional file 1 Figures ***S1 ***and ***S2***. For most reads, both hits passed very high BLAST and coverage thresholds ("classified") and those with slightly lower BLAST scores ("remain") represent background contamination. Several hundred reads and some contigs showed very weak or no BLAST hits and there are some weak hits to known virus families. However, none were judged to be clear-cut candidates for novel viruses.

**Table 2:** Results from metagenomic sequencing.

Library	Type	Reads	Mean	Max	MBp
454	DNA	53,984	170 bp	397 bp	9.1 mb
Sanger	DNA affected twins	787	716 bp	950 bp	0.56 mb
Sanger	DNA unaffected twins	756			
454	RNA	305,191	195 bp	331 bp	59.5 mb
Sanger	RNA affected twins	762	720 bp	1412 bp	0.59 mb
Sanger	RNA unaffected twins	757			

**Table 3:** Removal of reads matching the human genome sequences.

Library	Type	Reads	Human reads screened	After screening
454	DNA	53,984	20,376	33,608
Sanger	DNA	787	246	541
Total	DNA	54,771	20,622	34,149
454	RNA	305,191	263,436	41,755
Sanger	RNA	762	450	312
Total	RNA	305,953	263,886	42,067

**Table 4:** miraEST assembly of non-human sequence reads.

Library	Contigs	Max/mean length	Reads/contig	Singletons	Max/mean length
DNA	1,875	1,679/214	6.15	17,640	396/184
RNA	4,541	2,779/350	7.22	6,374	330/191

**Figure 3:**
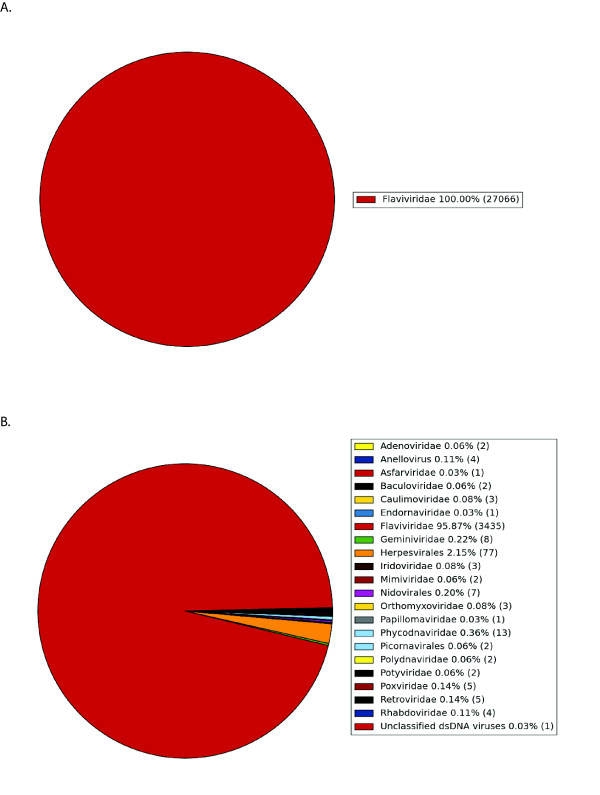
**Further classification of BLAST hits into virus families**. The sequences are 454 sequences from CFS patients classified with high confidence (panel A) and by closest homologue (panel B).

Notably, 29,463 454 reads and 7,105 contigs showed high BLAST identity with GBV-C. As expected for the RNA virus GBV-C, 99.5% of the reads came from the RNA (+RT) fraction (Figure 3). Similarly 1,354 reads and 162 contigs contained Hepatitis C virus sequences, almost entirely from the RNA fraction. These results confirmed the significant presence of these viruses in samples from the affected twins. Due to the efficient virus particle enrichment procedure used, it is highly likely that these sequences come from free virus particles and that one or more patients have chronic infection of these viruses.

### Confirmation in individual samples

#### GB virus C

Assessment of the individual samples using nested PCR showed that four samples from affected twins (8.9%) and zero from unaffected twins (0%) were positive for GBV-C. One affected twin had ICF and the rest had CFS. The first round PCR gave a visible product in all four positive cases indicating at least moderately high viral copy number. Detection of GBV-C in affected co-twins was slightly but significantly higher than chance expectations (using conditional logistic regression to account for paired sampling, likelihood ratio 5.54, df = 1, p = 0.019).

To assess GBV-C sequence diversity, 28,451 sequence reads from the RNA fraction matching the GBV-C genome were compared with the 23 complete GBV-C genome sequences found in Genbank. Using strict BLAST score criteria, the GBV-C samples in our data set were found to be quite diverse as regions from 19 of the complete genomes were represented in the dataset. However, 51% of the sequences (14,667 of 28,451) were divided between five different isolates in roughly equal numbers. GBV-C is known to vary extensively between isolates and the large diversity revealed here indicates that these four affected twins were infected by different isolates and that different variants are present in each individual.

#### Hepatitis C virus

A standard diagnostic serology test confirmed previously unrecognized hepatitis C infection in one affected twin. This discovery provides a plausible medical explanation for chronic fatigue in this individual.

## Discussion

We used an "unbiased" genomic technology to search for the presence of known and novel viruses that correlate with the clinical presence or absence of chronic fatiguing illness. Such searches have proven powerful for respiratory infections [14,15], and complement studies targeting specific infectious agents [13]. The general hypothesis we tested was that chronic fatigue was associated with on-going viremia. As we have argued elsewhere [12], the study of discordant monozygotic twins was optimal in controlling for potential biases particularly as samples were obtained from both twins at the same place and time.

The deep Roche 454 sequencing, combined with the efficient enrichment of virus particles, makes it likely that most viruses present in the serum of these individuals were detected. However, we did not detect any clear-cut signatures of novel viruses. For known viruses, the predominant finding was a slight but significant excess of detection of nucleic acid from GBV-C in 8.9% of affected twins and 0% of their unaffected co-twins (p = 0.019). Previously undetected hepatitis C virus infection was discovered in one affected twin. This individual was kept in these analyses as this is conservative and conforms to our prior intentions.

GBV-C (also known as hepatitis G virus) is an RNA virus and member of the Flaviviridae family with greatest homology to hepatitis C virus. It is transmitted via multiple modalities (e.g., vertically, sexually, and parenterally) [17]. GBV-C viremia is present in ~2% of healthy blood donors and 17% show evidence of past infection [18]. GBV-C infection is not known to cause any human disease [19] and co-infection might improve the course of HIV-1 disease [20]. A prior small study of 12 CFS cases and 21 controls concluded that chronic GBV-C infection was not associated with CFS [21]. The lack of GBV-C positive individuals among the unaffected twins is could at first glance be seen as surprising. However, we would statistically expect that one or two individuals would be positive, based on chance, and the result we obtained is therefore not unlikely.

There are several reasons why a chronic infection important to the etiology of chronic fatiguing illness could have escaped detection. For example, viral titers might be beneath the detection limit of our approach, the infection might be intermittently active and not during our sampling, and a salient infection might occur in body compartments or tissues where viral particles do not appear in blood. It is also possible that a salient infection occurred earlier in life, was cleared, but the infection sequelae are responsible for clinical state. Such infections, in the case of known viruses, can in many cases be detected via serology. Finally, it is possible that chronic fatiguing illness represents a similar clinical endpoint for multiple different disease etiologies (which may or may not be infectious in nature) and that etiological heterogeneity effectively lessens the probability of detection.

## Conclusions

Our results show a weakly significant difference between affected and unaffected twins in the cross-sectional prevalence of GBV-C viremia. Whether this is etiologically important or due to chance or bias is not clear. However, the possible connection between GBV-C and CFS deserves further study in larger samples.

## Methods

### Subjects

The protocol was approved in advance by the ethical review board at UNC-CH and the Karolinska Institutet and all subjects provided written informed consent. The parent study is described elsewhere [22-24], and we have previously shown that there were no differences in gene expression in peripheral blood in monozygotic twins discordant for chronic fatigue [12]. We screened ~61,000 individual twins from the Swedish Twin Registry for the symptoms of fatiguing illness. All twins were born in Sweden of Scandinavian ancestry. Of 5,597 monozygotic twin pairs where both were alive and had provided usable responses to CFS screening questions, we identified 140 pairs of twins who met preliminary inclusion criteria: born 1935-1985, classified as a monozygotic twin based on questionnaire responses [25], and discordant for chronic fatiguing illness (i.e., one twin reported substantial fatigue and the other twin was evidently well). A telephone interview using a standardized script was used to assess eligibility for participation. Twins who remained eligible both attended a half-day clinical assessment by a specially trained physician at the Karolinska Institutet in Stockholm. At this visit, a CFS-focused medical assessment was conducted that included standardized medical history, physical examination, and screening biochemical, hormonal, and hematological studies in accordance with international recommendations [1].

Of 140 monozygotic and preliminarily discordant twin pairs, one or both twins declined participation in 23 pairs, 25 pairs were concordant for CFS-like illness, and inclusion criteria were not met in 35 pairs (e.g., chronic fatigue had resolved or an illness that could explain fatiguing symptoms such as neoplasia had emerged). After excluding these 83 pairs, 57 pairs of twins attended the clinical evaluation sessions, and 10 pairs were found not to meet inclusion criteria (9 pairs were concordant for the presence or absence of chronic fatigue or a medical explanation was detected and 1 pair was dizygotic). Serum samples were unavailable for both members of 2 pairs. Zygosity was confirmed by genotyping 46 single nucleotide polymorphisms using two Sequenom iPlex panels.

The analysis sample consisted of 45 pairs of rigorously discordant and genetically proven monozygotic twins. Discordance was defined as one twin meeting criteria for either idiopathic chronic fatigue (ICF, 13 pairs) or CFS (32 pairs) [1,2] and the co-twin was required never to have experienced impairing unusual fatigue or tiredness lasting more than one month. Thus, all affected twins were required to have current, long-standing (≥6 months), medically unexplained fatigue associated with substantial impairment in social and occupational functioning and the unaffected co-twins were effectively well.

### Biological sampling

Biological sampling was standardized by having samples drawn from both members of a twin pair at the same place and time (~0900) after an overnight fast. We required that all subjects be in their usual state of health on the day of sampling (i.e., no acute illness or recent exacerbation of a chronic illness). It was neither practical nor ethical to study subjects medication-free, but we delayed assessment if there had been a recent significant dosage change. Peripheral venous blood was drawn using sterile technique.

### Viral library preparation and sequencing

Serum samples from 45 pairs of affected and unaffected monozygotic twins were available for this study. Sample preparation for library construction was as described previously [14] and, briefly, consists of viral particle recovery and nucleic acid extraction, followed by amplification and cloning of viral nucleic acid. Serum samples (200 μl) from the affected twins were pooled separately from their unaffected co-twins. Serum pools were then filtered either through 0.22 μm or 0.45 μm membrane filters (Millipore) and virus particles were concentrated by ultracentrifugation (41,000 rpm for 1.5 h at 4°C in a Beckman SW41 rotor). Exogenous nucleic acids were removed by DNaseI and RNaseA treatment followed by extraction of viral DNA (Qiagen) or RNA (Trizol, Invitrogen). First strand synthesis was carried out with a random primer containing an EcoRV site plus exonuclease negative Klenow polymerase (Promega) for DNA and Superscript II reverse transcriptase (Invitrogen) for RNA. Second strand synthesis for the above reactions was carried out with exonuclease negative Klenow polymerase (Promega). These were then amplified with AmpliTaq Gold polymerase (Applied Biosystems) and a primer complementary to part of the random primer used in first strand synthesis. PCR products were purified, digested with EcoRV, subjected to gel electrophoresis, and bands 500 bp - 5 kb were extracted from the gels. Blunt-ended PCR products were then cloned into pCR-blunt (Invitrogen) and transformed into TOP10 chemically competent cells for sequencing of clones. The library was then verified using conventional Sanger sequencing with DYEnamic Dye Terminator kits and a Megabace 1000 sequencer (GE Healthcare). Gel-purified blunt-ended PCR products (1.25-1.35 μg) were subjected to ultra-deep sequencing using the 454 FLX chemistry and sequencer (Roche) according to the manufacturer's instructions at the time.

### Computational analysis

Even though enriched for viruses, most of the sequenced samples contained a large fraction of human reads. For the purpose of analyzing the viral content of the data, human reads can be removed from the samples before assembly without affecting the results. The benefits of removing human sequences pre-assembly include a heavily reduced assembly time and a reduced risk of mis-assembly. Most human reads are highly homologous to human database sequences and can be identified with MegaBLAST [26]. Multiple NCBI databases (i.e., EST-Human, Human Genomic, and Human Genomic Transcripts) [27] were used to identify human reads. Highly repetitive human reads identified by MegaBLAST were also discarded. The remaining overlapping reads were then assembled into contigs using miraEST [28] which can perform a hybrid assembly using both Roche/454 and traditional Sanger sequences.

Before attempting to classify the contigs and singletons, highly repetitive sequences were eliminated using the DUST algorithm [29]. Remaining sequences were classified through a protocol of database alignment searches using NCBI BLAST [30]. Alignment search tools trade speed for sensitivity: for metagenomic datasets, efficient identification of more distantly homologous matches is accomplished using progressively more sensitive searches (rather than a single sensitive search). Progressive searches were performed using MegaBLAST against NCBI NT, then using BLASTn against NCBI NT, and finally using BLASTx against NCBI NR. For example, for a set of Roche/454 RNA reads, 70% of the remaining sequences were classified in the first step leaving far fewer data for the more time-consuming second and third steps. Sequences were then classified using the closest homologue defined by the alignment searches. Two main categories were built: classified sequences that are highly similar to a database sequence (> 90% identity with >70% query coverage) and "remainder" sequences that may contain new findings. Each category was split into taxonomy divisions and the virus division was further split into suitable virus subgroups to aid analysis.

### Total nucleic acid extraction and PCR of individual serum samples

Serum samples (400 μl each) were used for total nucleic extraction using the Virus Mini M48 kit (Qiagen) according to the manufacturer's instructions. The automated extraction process was carried out in a Qiagen Biorobot M48.

Presence of GBV-C virus in the samples was confirmed by nested PCR with primers specific for the 5' UTR of virus RNA [31]. First-round, one-step RT-PCR consisted of 1× AmpliTaq buffer (Applied Biosystems), 2 mM MgCl_2_, 200 μM dNTP mix, 0.4 μM of each primer GBV-F1 (5' CGGCCAAAAGGTGGTGGATG 3') and GBV-R1 (5' CACTGGTCCTTGTCAACTCG 3'), 5 μl of sample, 4 units AMV RT (Promega), 16 units of RNasin (Promega) and 1 unit of AmpliTaq DNA polymerase in a 50μl reaction. Cycling conditions were: 42°C for 60 min, and 35 cycles of 95°C for 1.5 min, 55°C for 2 min, 72°C for 3 min. The expected product size was 299 bp. Five μl of the first round reaction was used for a second round PCR reaction, which consisted of 1× AmpliTaq buffer, 2 mM MgCl_2_, 200 μM dNTP mix, 0.4 μM of each primer GBV-F2 (5' GGTGATGACAGGGTTGGTAG 3') and GBV-R2 (5' GCCTATTGGTCAAGAGAGACAT 3'), 1.25 U AmpliTaq DNA polymerase in a 50μl reaction. Reaction conditions were 94°C for 10 min, 35 cycles of 94°C for 30 s, 60°C for 30 s, 72°C for 1 min, and 72°C for 10 minutes. The expected PCR product size was 251 bp.

The diversity of GBV-C reads were compared against a database of complete GBV-C genome sequences from Genbank (23 sequences) using BLAST. A sequence was classified as similar to a certain isolate if the BLAST hit e-value was < 10^-20 ^and if the top hit was at least 100 times more significant than the second hit.

## Authors' contributions

All authors reviewed and approved the final version of the manuscript. AJ and BE were responsible for the clinical evaluations. BA, TA, and SG conducted the DNA and RNA analyses and verification experiments. FL and BP performed bioinformatics analyses. NLP supervised the fieldwork in Sweden. PFS, NLP, and BA designed the study and obtained funding. PFS and BA wrote the manuscript.

## Financial Disclosures

The funders had no role in study design, data collection and analysis, decision to publish, or preparation of the manuscript. In the interests of full disclosure, Dr. Sullivan reports receiving unrestricted research funding from Eli Lilly for genetic research in schizophrenia. The other authors report no conflicts.

## Supplementary Material

Additional file 1 **Supplemental figures**. contains the two supplemental figures referenced in the text. Click here for file
