# Supplementary material for: An unbiased metagenomic search for infectious agents using monozygotic twins discordant for chronic fatigue
Source: BMC Microbiol. 2011 Jan 2;11:2. doi: 10.1186/1471-2180-11-2 (PMC3022642; doi:10.1186/1471-2180-11-2)

***An unbiased search for infectious agents in monozygotic twins discordant for chronic fatigue***

Sullivan .. Andersson

Additional file 1, Figures S1 and S2

***Figure S1. Virus Classifications, reads***

A. Virus family assignments, Sanger reads, CFS cases, DNA fraction, Classified


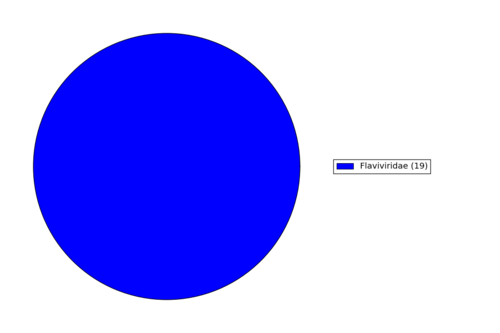


B. Virus family assignment, Sanger reads, CFS cases, DNA fraction, remain


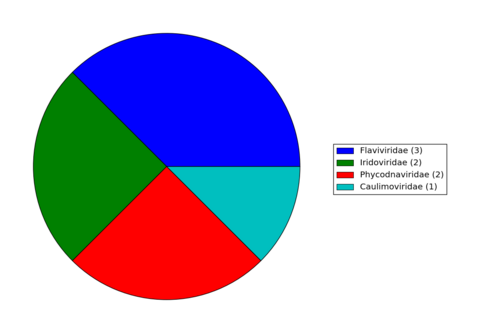


C. Virus family assignment, Sanger reads, CFS cases, RNA fraction, classified


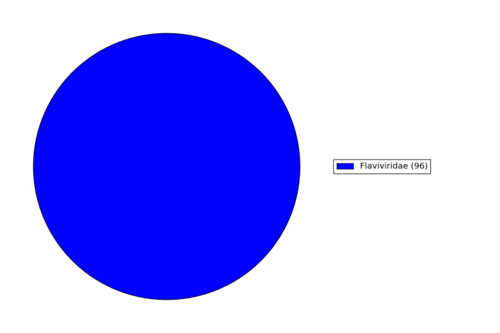


D. . Virus family assignment, Sanger reads, CFS cases, RNA fraction, remain


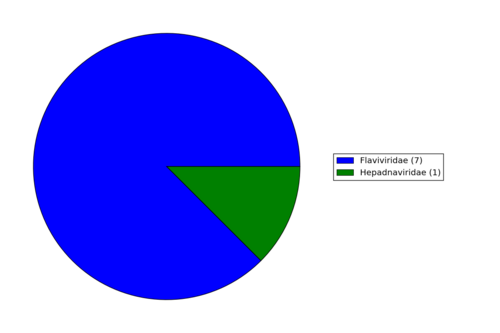


E. Virus family assignment, 454 reads, CFS cases, DNA fraction, classified


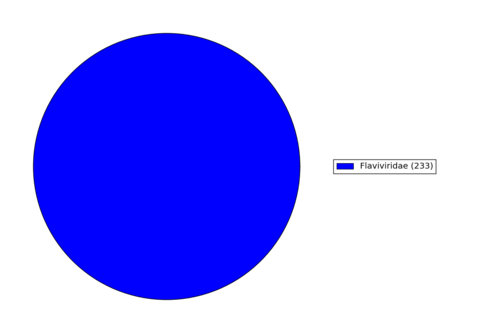


F. Virus family assignment, 454 reads, CFS cases, DNA fraction, remain


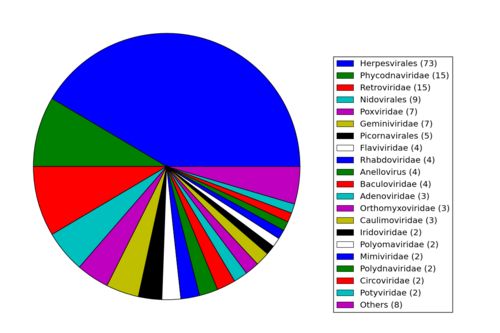


G. Virus family assignment, 454 reads, CFS cases, RNA fraction, classified


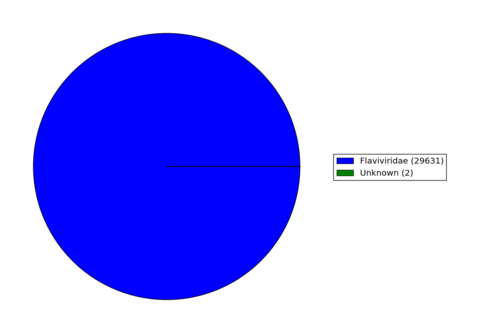


H. Virus family assignment, 454 reads, CFS cases, RNA fraction, remain


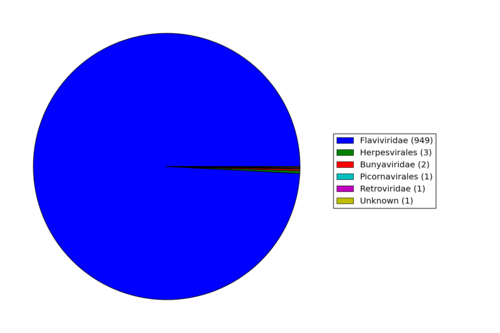


***Figure S2. Virus classifications, contigs***

A. Virus family assignment, all contigs, CFS cases, DNA fraction, classified


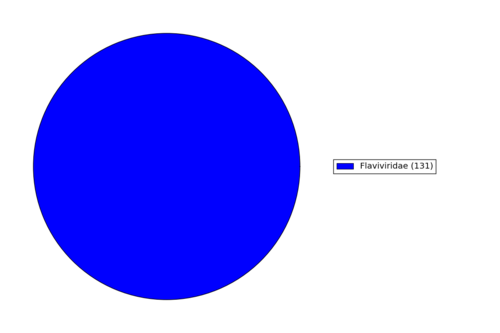


B. Virus family assignment, all contigs, CFS cases, DNA fraction, remain


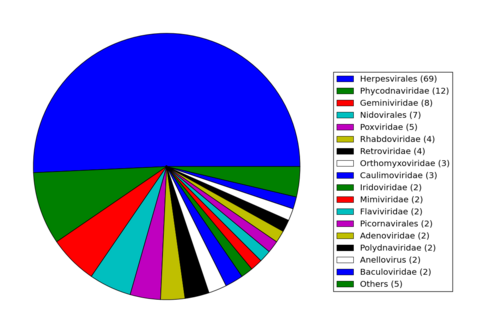


C. Virus family assignment, all contigs, CFS cases, RNA fraction, classified


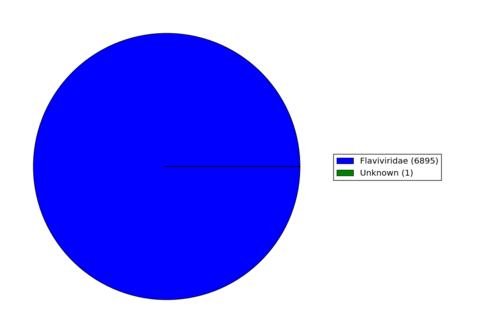


D. Virus family assignment, all contigs, CFS cases, RNA fraction, remain


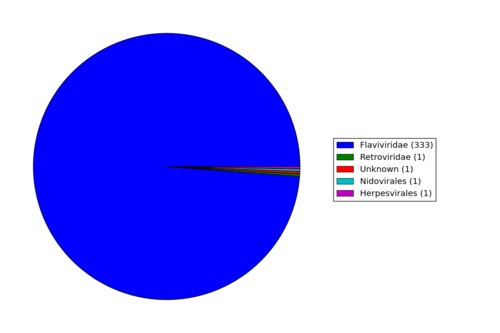

Supplement: Additional file 1 — Supplemental figures. contains the two supplemental figures referenced in the text. [file 1471-2180-11-2-S1.DOC]
